# NeuroAIreh@b: an artificial intelligence-based methodology for personalized and adaptive neurorehabilitation

**DOI:** 10.3389/fneur.2023.1258323

**Published:** 2024-01-19

**Authors:** Ana Lúcia Faria, Yuri Almeida, Diogo Branco, Joana Câmara, Mónica Cameirão, Luis Ferreira, André Moreira, Teresa Paulino, Pedro Rodrigues, Mónica Spinola, Manuela Vilar, Sergi Bermúdez i Badia, Mario Simões, Eduardo Fermé

**Affiliations:** ^1^Department of Psychology, Faculty of Arts and Humanities, University of Madeira, Funchal, Portugal; ^2^NOVA Laboratory for Computer Science and Informatics, Caparica, Portugal; ^3^Agência Regional para o Desenvolvimento da Investigação, Tecnologia e Inovação, Funchal, Portugal; ^4^Department of Informatics Engineering, Faculty of Exact Sciences and Engineering University of Madeira, Funchal, Portugal; ^5^Faculty of Psychology and Educational Sciences, University of Coimbra, Coimbra, Portugal; ^6^Center for Research in Neuropsychology and Cognitive and Behavioral Intervention, Coimbra, Portugal

**Keywords:** neurorehabilitation, virtual reality-based activities of daily living simulations, artificial intelligence in health, profile dynamics, knowledge representation and reasoning applications, stroke

## Abstract

Cognitive impairments are a prevalent consequence of acquired brain injury, dementia, and age-related cognitive decline, hampering individuals' daily functioning and independence, with significant societal and economic implications. While neurorehabilitation represents a promising avenue for addressing these deficits, traditional rehabilitation approaches face notable limitations. First, they lack adaptability, offering one-size-fits-all solutions that may not effectively meet each patient's unique needs. Furthermore, the resource-intensive nature of these interventions, often confined to clinical settings, poses barriers to widespread, cost-effective, and sustained implementation, resulting in suboptimal outcomes in terms of intervention adaptability, intensity, and duration. In response to these challenges, this paper introduces NeuroAIreh@b, an innovative cognitive profiling and training methodology that uses an AI-driven framework to optimize neurorehabilitation prescription. NeuroAIreh@b effectively bridges the gap between neuropsychological assessment and computational modeling, thereby affording highly personalized and adaptive neurorehabilitation sessions. This approach also leverages virtual reality-based simulations of daily living activities to enhance ecological validity and efficacy. The feasibility of NeuroAIreh@b has already been demonstrated through a clinical study with stroke patients employing a tablet-based intervention. The NeuroAIreh@b methodology holds the potential for efficacy studies in large randomized controlled trials in the future.

## 1 Introduction

According to the World Health Organization, dementia and stroke are among the leading causes of disability and dependency. By 2050, the percentage of older people should increase by 35%, which raises the number of people at risk of developing dementia from any etiology (1). Up to 53.7% of all cases of dementia are assumed to be due to Alzheimer's disease (AD), while 15.8% are considered to be due to Vascular Dementia (VD) (2). With, so far, no effective pharmacological treatment found, the increase of older adults with cognitive impairments makes it urgent to deliver adapted/personalized neuropsychological interventions in individuals with Mild Cognitive impairment (MCI), a clinical condition that increases the risk of developing dementia in 38% (3); and stroke, which likelihood to develop VD is estimated to range from 36 to 67% (4).

The Neuropsychological Assessment (NPA) is a comprehensive evaluation of an individual's cognitive, emotional and behavioral functions, typically conducted by a neuropsychologist. It involves a variety of instruments to assess different aspects of brain function, such as memory, attention, language, and executive functioning. NPA is often performed in clinical settings to diagnose and treat conditions such as acquired brain injuries, neurodegenerative diseases and psychological disorders. NPA is essential to determine a patient's cognitive profile. An Assessed Cognitive Profile (ACP) refers to the formal measurement of an individual's cognitive abilities and functioning. This profile includes information about various aspects of cognition (memory, attention, language, and executive functioning) and outlines an individual's strengths and weaknesses in these domains. The ACP is valuable for various purposes, including diagnosing cognitive impairments, monitoring changes over time and/or guiding intervention strategies, making it a crucial component in healthcare and education settings. However, its paper-and-pencil methodologies have fallen reliant on labor-intensive procedures of data collection that provide relatively data-poor estimates of human behavior despite the rapid technological advances in other healthcare fields (5). Integrating technology, namely artificial intelligence (AI) methodologies, into NPA practices has tremendous potential to advance the field faster in numerous areas, such as neurorehabilitation (6).

Technology-based assessment and rehabilitation methods with high ecological validity, particularly those based on the use of Virtual Reality (VR), have been shown to lead to increased outcomes in neurorehabilitation (7). One reason for this could be the fact that VR-based methods allow incorporating cognitive tasks within the simulation of Activities of Daily Living (ADLs) and the creation of well-controlled environments oriented toward the needs of patients (8–10). Reh@City, a VR-based neurorehabilitation tool, is an example where memory, attention, language, and executive functions tasks are integrated into the performance of several ADLs (11). A randomized controlled trial with stroke participants who underwent rehabilitation with the Reh@City revealed a significant impact on cognitive and functional domains compared to equivalent standard paper-and-pencil tasks (12).

Recently, a questionnaire was delivered to healthcare institutions in Portugal to understand the actual perspective of health professionals on using technologies for cognitive rehabilitation (CR) (13). Data from 116 participants showed that health professionals mostly use games, puzzles, and paper-and-pencil tasks. Concerning the profile of patients undergoing CR, dementia and stroke were reported as the main conditions being addressed, and most patients were above 60. Results indicated that technologies are not yet widely used by health professionals in CR sessions, with most participants (65.5%) reporting no experience with CR technologies. The most mentioned barriers were the nonexistence of technologies at the institution and the lack of qualified human resources to support them.

The limited adoption of computer-based NPA and neurorehabilitation (14) might be explained by the fact that, rather than incorporating many of the advances in neuroscience or computer science, most test developers redesign paper-and-pencil methods for administration on the computer (5). Although digitizing current tests certainly has advantages over its analog tests, these could be leveraged far more effectively if development efforts also focused on capturing more behavioral data and increasing the ecological validity of tests (15).

Over the last decades, AI capabilities have grown exponentially, and, in recent years, it has become ubiquitous. It is everywhere, from cars to smartwatches, from smart TVs to the operation room in advanced hospitals. Martens et al. (16) identified in their work that, as performance increases, the readability decreases. They ordered AI systems from less performance to most performance: Rule-based Systems, intrinsically linear models, and Artificial Neural Networks/Support Vector Machines. The application of Machine Learning (ML)-based methods in healthcare is also rapidly evolving with practical implications in the prevention, diagnosis, treatment, and prognosis of specific clinical conditions (17, 18). To the best of our knowledge, only three neurorehabilitation platforms are using an AI-driven approach to adapt and personalize training sessions: the Guttman Neuropersonal Trainer (GNPT) (19), the Neuro-World (20), and the Brain Training System (BTS) (21, 22).

The GNPT consists of a tele-CR platform for patients diagnosed with Acquired Brain Injuries (ABI), aiming to provide neuropsychologists services beyond the clinical setting and increasing the personalization, duration, and intensity of the neurorehabilitation process (19). This platform encompasses telemedicine services and AI for knowledge extraction (e.g., data mining, collaborative environments, and automatic system adaptation to patients' performance). The CR personalization process begins with the performance of a baseline NPA; then, the assessment results are stored in the GNPT system and used to define the patients' cognitive profile. The system proposes a Cognitive Training (CT) therapeutic plan based on this profile. The adjustment of the therapeutic plan (i.e., type of CT tasks and difficulty levels) is performed automatically by the system, according to the patient's performance (23). Regarding rehabilitation content, this platform comprises 95 computerized exercises, targeting several cognitive functions (19).

Concerning the Neuro-World, it is comprised of a set of six mobile games designed to challenge visuospatial short-term memory and selective attention (20). The approach allows self-administration of assessment and remote monitoring of the patient's cognitive status (CS). This process happens by analyzing the patient's game performance and estimating the Mini-Mental State Examination (MMSE) results through ML algorithms. A longitudinal study with 12 stroke survivors with mild cognitive deficits demonstrated that the Neuro-World could estimate the MMSE scores with a low normalized root mean square error (5.75%). An interesting contribution of this work is that assessment and rehabilitation can be combined in the same tool.

Lastly, the BTS uses an algorithm that automatically selects and schedules cognitive training exercises (21, 22). The difficulty level of the exercises is generated around the ACP of the participant, which is updated as the participant progresses. The system uses a scoring system to compare performances in different exercises that are merged according to the same cognitive domain level. A supervision process based on “red flags” is activated whenever the system detects user engagement, compliance, or adherence issues.

The existing AI-driven cognitive rehabilitation platforms have several limitations in common, namely: (1) Limited transfer effect as the cognitive skills acquired through these platforms may not always generalize to real-world tasks, as they often lack ecological validity; (2) Reduced engagement and motivation, as some users may find neurorehabilitation tasks repetitive or uninteresting; (3) Lack of clinical supervision, which could potentially lead to suboptimal progress or user frustration; (4) Focus on a limited range of cognitive domains and; (5) The inappropriate personalization due to a one-size-fits-all approach may not be tailored to an individual's specific ACP.

Here, we present NeuroAIreh@b, a new cognitive profiling and training methodology that uses AI to maximize neurorehabilitation prescription personalization and adaptation. NeuroAIreh@b is being developed within a multidisciplinary environment, combining different expertise fields such as neuropsychology, computer science, game design and AI for health. Here, we will describe the methodology followed to address the challenges posed by the scientific literature in the field and neurorehabilitation clinicians. Specifically, we will explain how we: (1) create an optimal cognitive profile by aggregating the NPA instruments results (according to empirical input from neuropsychology experts) and then (re)calibrate them with the support of ML algorithms; (2) design and developed CTTs that can be prescribed according to the patient's ACP, training objectives and performance in previous CTTs; and (3) adapt the CTTs from session to session according to the patient's performance through the theoretical framework of dynamics of profiles developed in (24), based on Belief Revision (BR) theory (25, 26).

## 2 Methods

### 2.1 The framework and its challenges

Neurorehabilitation is the most effective approach to address cognitive deficits (27). However, current tools are (a) challenging to adapt to every patient since they demand the application of an extensive battery of NPA instruments, which results are interpreted manually and often prone to errors in the selection of CTTs, (b) have a high implementation cost, since they involve several sessions performed in clinical environments by neuropsychologists and (c) session to session adaptation to the patient performance is not always performed, which may limit the rehabilitation potential and motivation of the patient.

**Figure 1:**
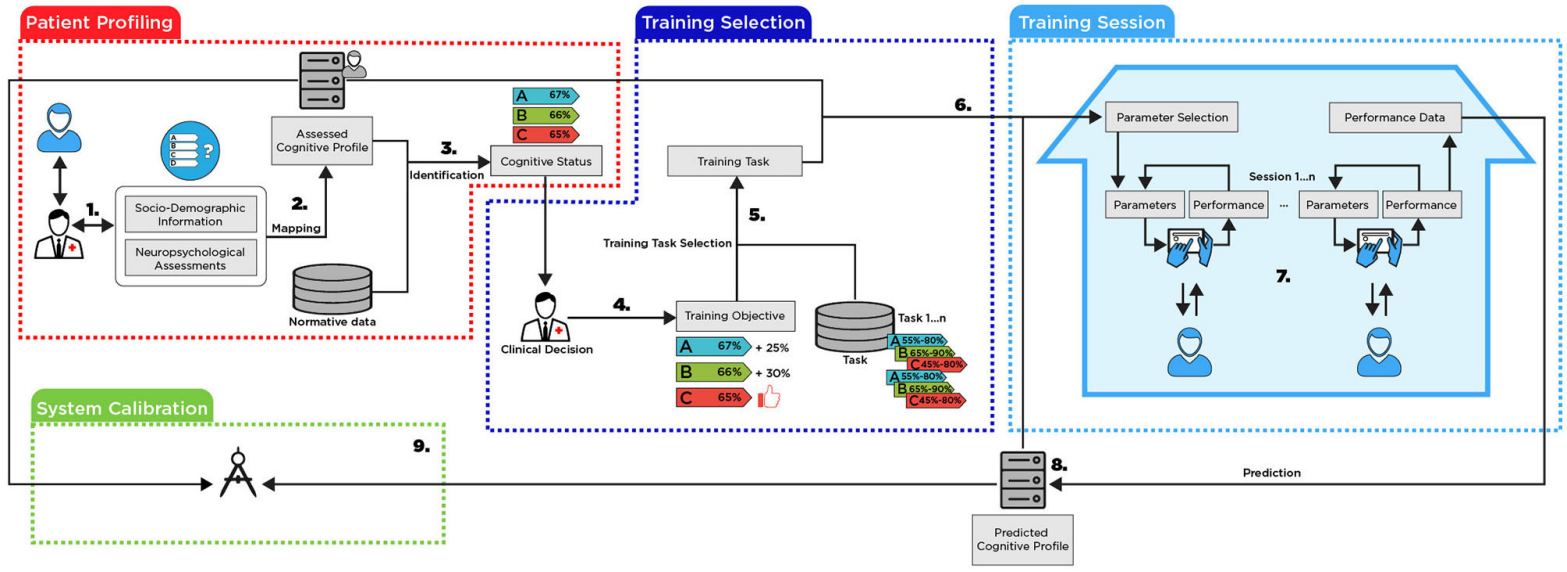
The proposed framework.

To address these main limitations, we developed a framework for personalized and adaptive delivery of neurorehabilitation that can be divided into four different components as indicated in Figure 1. In this section, we will describe, at a high level, the processes to be performed in each framework component (numbers 1–9 in the Figure 1) and the challenges involved in its construction.

**The patient profiling:** this component aims to create a multi-dimensional patient profile that integrates several NPAs to determine a baseline CS.

1. Neuropsychologists use validated NPA instruments to assess patients' cognitive functioning. An NPA instrument is a standard part of integrated medical care and is necessary to prescribe and evaluate (in terms of efficacy) rehabilitation procedures. A comprehensive NPA aims: (i) to measure and assess cognitive abilities, ADLs performance, personality traits, and emotional and behavioral functioning in light of the premorbid functioning of the patient; (ii) to quantify the nature and severity of cognitive and functional deficits, analyzing the symptoms and signs present in the context of the structural and functional integrity of brain functioning, to differentiate normal and pathological cognitive decline (iii) to define a baseline level of performance in cognitive, functional, and emotional functioning domains, which can be examined in a longitudinal registry, through repeated evaluations, thus enabling monitoring of the clinical evolution of the patient, in terms of response to interventions (e.g., CT, psychotherapy, pharmacological therapy) or disease's progression; (iv) to identify personal resources and preserved functions that are useful for planning and implementing compensatory intervention procedures.
  **Challenges:** To represent profiles, it is necessary to define a formal language. This language will be used for representing the profiles, for expressing their properties, for computing metrics about them and for determining their dynamics. After explaining the general structure, we must identify which cognitive, functional, behavioral, and emotional domains should be considered when creating patients' profiles. Also, it is necessary to specify relevant NPA for a comprehensive and multidimensional evaluation of these domains and determine which socio-demographic information (SDI) to consider for profiling purposes.
2. By aggregating the different NPAs with the SDI of the patient, the system creates an Assessed Cognitive Profile (ACP).
  **Challenges:** To the best of our knowledge, there is a gap in the literature on integrating data from multiple and heterogeneous NPAs and consolidating it into a consistent cognitive profile.
3. The ACP itself is not sufficient to determine the CS of the patient. It must be compared with the normative data available for each NPA tool.
  **Challenges:** The normative data for each NPA may be provided for different clinical conditions and are often separated by socio-demographic groups. As well as in step 2, this needs to be aggregated and compared to the consolidated profile with an objective and quantifiable distance metric.

**The training selection:** After determining the patients' CS, which gives information about the preserved and impaired domains, neuropsychologists must define the most appropriate CTTs for neurorehabilitation.

4. Based on the patient's CS, neuropsychologists determine specific training objectives for each patient, i.e., in which cognitive domains the rehabilitation training must be focused on to regain or compensate for lost cognitive abilities and functional independence.
  **Challenges:** The ultimate goal of neurorehabilitation is to help patients to regain independence and autonomy in their ADLs. It can be difficult to establish objectives on a system that will mostly accept numerical values when the objectives are usually set subjectively. The system should be able to perform this translation.
5. After establishing the training objectives and the set of available CTTs in the system, NeuroAIreh@bab will compute which tasks are most appropriate for the training. This is possible since each task has its own profile, i.e., one that details which cognitive domains are required to perform it and how the task's difficulty can be parameterized for the different cognitive domains.
  **Challenges:** Each task must include information about which cognitive domain it trains. Additionally, it should include constraints regarding the minimal and maximal values for a particular cognitive domain of the suitable patient profile. The set of selected tasks must be optimally provided with the training objective (considering that the number of selected tasks is also limited given the amount of time and number of sessions assigned to the CT program).
6. By combining the initial profile and CTTs, the system establishes the initial parameters for the tasks. These initial parameters determine the task's difficulty according to the different cognitive domains.
  **Challenges:** Combination is not trivial, and it is based on the tuple ≪task, the value of the cognitive domain in the profile, and associated difficulty≫ that will be adjusted in the system using ML techniques (see System Calibration).

**The training session:** This part describes how a patient would perform a CT session from the NeuroAIreh@b.

7. The patient can perform his prescribed training sessions at the clinic or at home. Each training session consists of a set of predefined tasks to be executed on a tablet or a personal computer. The NeuroAIreh@b itself will calculate the performance of the patient at each iteration and will redefine its difficulty by changing its parameters to maintain a patient score in a task between 50 to 70% of success to avoid both boredom and frustration, keeping the patients challenged and engaged (28).
  **Challenges:** To maintain the task score between 50 and 70%, the system must establish a relation between the scores in the different tasks, the task parameters, and the resulting difficulty for each cognitive domain of the patient profile.
8. After a complete training session, the system aggregates the performance in all tasks and estimates if there was evolution or involution in the different cognitive domains of the patient's profile, defining a *predicted cognitive profile*.
  **Challenges:** Defining the predicted cognitive profile involves multiple challenges. Given a profile representation, defining the profile dynamics when new information is provided is necessary. The system must perform minimal changes in the profile to accommodate the new information. This minimal change requires applying belief revision techniques adapted to the profile representation languages mentioned in Step 1.

**The system calibration:** This part describes the system's calibration when comparing the predicted profile with newly acquired data.

9. A new NPA is performed when a patient ends a training session and the cycle restarts. The newly assessed cognitive profile is compared with the predicted cognitive profile of the system. If they differ, the system analyzes the possible causes of the divergence and (re)calibrates the system adequately.
  **Challenges:** The divergences can have different origins: (1) a wrong prediction in step 8; (2) a non-accurate model of the relationship between tasks and cognitive domains in step 5; or (3) a suboptimal adjustment of the parameters in step 7.

#### 2.1.1 The role of artificial intelligence

As previously explained, the implementation of the proposed framework entails several challenges. This subsection briefly describes which parts and AI techniques we use to address the challenges.

The first resort to AI appears in the creation of the ACP [see (2) in Figure 1]. Here, we distinguish two different phases: In order to start with no data, a focus group of six experts in neuropsychological assessment and rehabilitation made an empirical analysis of the NPAs to aggregate them by cognitive domain. This aggregation (a weighted sum) was later checked by using belief merging and judgment aggregation procedures [for an overview, see (29, 30)] and compared with correlations between NPAs, established using ML techniques with available data for Alzheimer's disease, which also involves cognitive impairment. For the second phase, with a fully operational system, and after collecting enough data, we will calibrate the weight assigned to each NPA regarding each cognitive domain and subdomain by ML techniques. All this process is explained in detail in Section 2.2 (The Profiling Challenge). The same approach is used to aggregate the relation between each NPA and the normative data to obtain a consolidated CS.

The second appearance of AI methodology is to optimize the process of CTTs selection from the CTT repository [(5) in Figure 1]. This optimization is explained in detail in Section 2.4.1.

The next resort to AI appears for deciding the difficulty level of a CTT during a training session [(7) in Figure 1]. The CTT parameters must be adapted so that the patient obtains a performance between 50 and 70%. This adaptation is better explained in Section 2.4.2.

The overall training performance comparison along the 12 sessions intervention will be the input for calculating the Predicted Cognitive Profile (PCP) [(8) in Figure 1]. The process of relating the CTTs' difficulty and cognitive domains is not linear, mainly because a CTT trains multiple cognitive domains. It can be challenging to differentiate how much of an obtained performance relates to each specific domain. To create the PCP, the performance of the patient in the training session is transformed into a new entity described in a sentence in formal language (see Section 2.2.1). This constitutes an input for an update function that will actualize the profile, making a *minimal change* to incorporate the new information. The algorithm for this update is based on the theoretical framework for updates of profiles developed in (24).

Finally, the last AI challenge is to calibrate the system. As mentioned in Challenge 9, the divergences can have different origins. In this case, we will collect all the data to identify, via ML approaches, the origin of the divergences.

### 2.2 The Profiling Challenge

The Profiling Challenge corresponds to Steps 1, 2, and 3 of the framework illustrated in Figure 1.

#### 2.2.1 The profile's structure

In this subsection, we identify which aspects of cognitive, functional and emotional domains are relevant to include in the patient's profile and which NPA instruments are representative of those aspects. We start by defining a formal profile.

Definition 2.1. (24) A profile *P* is a tuple ≪*label*_1_, …, *label*_*n*_≫, where *label*_*i*_∈ℕ_0_.

Informally, each element of the tuple of a profile is a characteristic that assumes a finite number of possible values. We have used natural numbers for the content of each *label*_*i*_. However, it is easy to change the definition to use linguistic labels; for instance, if *label*_1_ represents the marital status, we can use “single/married/separated/widowed”, etc., as possible values.

A simple example of a profile structure is ≪age, weight, height≫ and a possible profile is John = ≪20, 80, 178≫.

The next step is to define a formal language for expressing the profile properties, for computing metrics about it and for defining its dynamics.

Definition 2.2. (24) A *profile language* is a finitary language L, defined in the following way:

*X* is a term if and only if:

1. *X* is a label.
2. If *X* is a term, then −*X* is a term.
3. If *X* and *Y* are terms, then *X*+*Y* is a term.

An atom is an expression of one of the following forms:

1. *X* = *n*,
2. *X*<*n*,
3. *X*>*n*,

where *X* is a term and *n*∈ℕ_0_. A well-formed formula (wff) is defined as:

1. An atom is a wff
2. If *X* is a wff, then ¬*X* is a wff
3. If *X* and *Y* are wff, then *X*∧*Y* are wff

where ¬ (negation) and ∧ (conjunction) are the classical negation and conjunction connectives.

≤, ≥ are defined in the usual way, as well as the classical connectives ∨ (disjunction), → (implication) and ↔ (equivalence). Backing to the previous example, “weight ≥90∧ height ≤ 180” is a wff.

After defining the profile structure and language, the next step is to define the contents of a profile in NeuroAIreh@b. Therefore, an integration of the relevant NPA instruments, with different weights per domain and subdomains, is essential for a comprehensive evaluation of cognition (see Table 1). For example, screening tests, such as the Montreal Cognitive Assessment (MoCA) (31), are brief multidomain screening instruments to identify cognitively at-risk patients requiring a more comprehensive evaluation. For example, in a domain and subdomain analysis, we have identified the following NPA dimensions in the MoCA: general cognition; orientation; immediate verbal memory; executive functions (namely, working memory, processing speed, verbal fluency and inhibition and visuoconstructive capacity); language (such as comprehension and expression) and sustained attention.

**Table 1:** Combination of the NPA instruments according to the cognitive domains and subdomains assessed.

**General cognition**			**MoCA total 100% (50%)**		**CDR cognitive cluster 100% (50%)**	
**Orientation**			**CDR orientation 33,33% (72,73%)**		**MoCA temporal and spatial orientation 12,5% (27,27%)**	
Memory	Immediate	Verbal	FCSRT immediate memory **100% (68,57%)**	CDR immediate memory **33,33% (22,86%)**		MoCA delayed recall **12,5% (8,57%)**
		Visual	FCRey 3 min **100% (50%)**	WMSIII visual reproduction immediate recall **100% (50%)**	
	Delayed	Verbal	FCSRT delayed recall **100% (75%)**		CDR memory delayed recall **33,33% (25%)**	
		Visual	WMS-III visual reproduction delayed recall **100% (50%)**		WMS-III visual reproduction recognition **100% (50%)**	
Executive functions	Working memory		WAISIII digit symbol coding **50% (68,54%)**	MoCA digit in reverse **12,5% (17,13%)**	MoCA calculus **6,25% (8,57%)**	MoCA target detection **4,2% (5,76%)**
	Processing speed		WAISIII symbol search **50% (36,36%)**	WAISIII digit symbol coding **50% (36,36%)**	Phonemic and semantic verbal fluency **33,33% (24,23%)**	MoCA phonemic verbal fluency **4,2% (3,05%)**
	Verbal fluency		Phonemic and semantic verbal fluency **33,33% (88,81%)**		MoCA phonemic verbal fluency **4,2% (11,19%)**	
	Inhibition		Phonemic and semantic verbal fluency **33,33% (79,88%)**		MoCA target detection **4,2% (10,06%)**	MoCA phonemic verbal fluency **4,2% (10,06%)**
	Visuoconstructive capacity		FCRey copy **100% (50%)**		WMSIII visual reproduction total score **100% (50%)**	
Language	Comprehension		WAIS-III vocabulary **50% (100%)**			
	Expression		MoCA naming and repetition **12,5% (100%)**			
Attention	Divided		WAIS-III symbol search **50% (100%)**			
	Sustained		Toulouse-Piéron test **100% (81,33%)**	MoCA target detection **4,2% (3,42%)**	MoCA calculus **6,25% (5,08%)**	MoCA digit direct **12,5% (10,17%)**
Premorbid intelligence			WAIS-III vocabulary **50% (100%)**			
Functionality		Basic ADLs	IAFAI basic ADLs **100% (50%)**		CDR personal care **100% (50%)**	
	Instrumental – familiar ADLs	Instrumental – familiar ADLs **100% (50%)**		CDR home and hobbies **100% (50%)**	
	Instrumental – advanced ADLs	Instrumental – advanced ADLs **100% (33,33)**		CDR community affairs **100% (33,33%)**	CDR judgment and problem solving **100% (33,33%)**	
	Cognitive deficits perceived impact		PRECiS or SMC* **100%**			
Depressive symptomatology			GDS-30 or BDI-II* **100%**			

The normalized values are presented between (). *Selection according to the patient's age.

We considered the demographic variables, such as education and age for analyzing the NPA instruments results. The rationale for this option recognizes the impact of these variables in explaining the variance of results and defining the test scores' normative benchmarks. From a rehabilitation perspective, it is essential to consider other variables such as household and occupation.

The results obtained with a comprehensive battery of NPA instruments provides a baseline of impaired cognitive function(s), which helps to define the duration and type of neurorehabilitation that needs to be performed. For example, executive functions deficits, specifically in inhibition, planning and monitoring, demand intervention programs focused on executive functioning. Additionally, the results obtained in the memory tests contribute to personalizing and adapting CR sessions to involve the use of a calendar and notepad, warnings, teaching and training of mnemonics, face-name associations, improvement of episodic memory, semantic memory, autobiographical memory, and visual memory.

Additionally to the MoCA screening instrument, we have established the following multidimensional battery of NPA instruments to create a profile in the NeuroAIreh@b: the Clinical Dementia Rating (CDR) (32); the Subjective Memory Complaints (SMC) (32); the Free and Cued Selective Reminding Test (FCSRT) (33); the Visual Reproduction from the Wechsler Memory Scale (WMS-III) (34); the Semantic and Phonemic Verbal Fluency Tests (35); the Toulouse-Piéron test (36); the Digit Symbol Coding, the Symbol Search and the Vocabulary Subtests from the Wechsler Adult Intelligence Scale Subtests (WAIS-III) (37); the Rey-Osterrieth Complex Figure Test (38); the Adults and Older Adults Functional Assessment Inventory (IAFAI) (39) or the Patient-Reported Evaluation of Cognitive State (PRECiS) (40); the Geriatric Depression Scale-30 (GDS-30) (41) or the Beck Depression Inventory-II (BDI-II) (42); and the World Health Organization Quality of Life — Old (WHOQOL-OLD) (43) or the Quality of Life after Brain Injury (QOLIBRI) (44). This selection was made according to the following criteria: (1) NPA instruments that are standard and widely used in Portuguese clinical and research contexts; (2) adequate NPA instruments with specificity for detecting impairments in the cognitive, functional and emotional dimensions of stroke, MCI and dementia clinical populations; and (3) NPA instruments with normative data for the Portuguese population.

#### 2.2.2 Aggregation of neuropsychological assessments

To create the ACP, we established a bridge between the different NPAs and the SDI of the patient. To the best of our knowledge, there is a gap in the literature regarding the integration of data from multiple and heterogeneous NPA instruments to create an ACP.

As stated above, in the initial phase we did not have enough data on stroke, MCI and dementia patients to use an ML approach and quantify the contribution of each NPA to the different dimensions of the profile. Therefore, we developed the following strategy: (1) A focus group of 6 neuropsychological assessment and rehabilitation experts defined a general formula for aggregating the NPAs, considering weights for the relation between NPA instruments and cognitive domains/subdomains (2) the NPA instruments were empirically aggregated, based on the expert's experience, and a first value for the weights was obtained, (3) the previous aggregation was pre-validated by using correlations obtained from patients with dementia and weights were readjusted, and (4) ML algorithms for future calibrations were defined.

#### 2.2.3 The general formula

We propose to map NPA instrument scores to a consolidated ACP in the interval between 0 and 100, and the Equation (1) is used for the *Mapping*. Note that it is formulated owing to no data.


(1) $$\begin{array}{l} ACP_{k}=\overset{n}{\underset{i=1}{\sum }}\overset{m}{\underset{j=1}{\sum }}Norm\left(NPA_{i}Dom_{j}\right).W_{kij} \end{array}$$

where, *m* is the number of times a *Dom* a NPA tool appears and *n* the number of NPA instruments. *ACP*_*k*_ is the cognitive domain *k* for this ACP, *NPA*_*i*_*Dom*_*j*_ is the domain/subdomain *j* of the NPA tool *i*, *Norm* is a normalization function, which interval is ranged between 0 and 100. Finally, *W*_*kij*_ is a weight value in the interval 0 to 1, where ∑*w*_*kij*_ = 1. The *ACP* for a patient is therefore defined as


(2) $$\begin{array}{l} ACP=\ll ACP_{1},\ldots ,ACP_{r}\gg . \end{array}$$

where *r* is the total number of domains/subdomains considered.

For example, if we use the weights for working memory (wm) provided on Table 1 we obtain:


$$Working\_Memory=\{\begin{array}{l} WaisIII\_DSC_{\text{wm}}*0.6854+\\ +MoCA\_digit_{\text{wm}}*0.1713+\\ +MoCA\_calculus_{\text{wm}}*0.0857+\\ +MoCA\_target_{\text{wm}}*0.0576 \end{array}$$

The *ACP* itself is not enough to determine the CS of a patient. To obtain it, we need to compare it with the Normative Data (ND). The ND is organized considering the SDI of the patient. If we interpret the ACP using the ND from all the NPAs considering the patient's SDI, we will get his/her CS. The next step is to solve how to contextualize this profile. We propose the following formula:


(3) $$\begin{array}{l} SCP_{k}=\overset{n}{\underset{i=1}{\sum }}Norm\left(ND_{i},SDI\right).w_{i} \end{array}$$

where *n* = number of NPA instruments. *SCP*_*k*_ is the CS for the domain/subdomain *k* from ND and SDI. *Norm* is a normalization function in which the interval ranges from 0 to 100. *ND*_*i*_ is the profile placement in the normative data of *k* for each NPA tool *i* used to calculate *ACP* and *SDI* is patient's SDI. Finally, *w*_*i*_ is the weight value of the ND *i* for the domain *k* in the interval 0 to 1, and the ∑*w*_*i*_ = 1.

First, it is essential to mention that we do not have access to the normative values for each NPA /task/question. These data are only available for the total and, in some cases, sub-totals of each NPA. A weight needs to be given for the pair ND-SDI to have the aggregated result of all NPA instruments involved when we do the sum. This pair provides the average performance on a specific NPA for someone that belongs to the same socio-demographic group (e.g., 65–70 years old, 12 years of schooling) as the patient. Since the MoCA ND values are available for the Portuguese population (45), we will use it as an example, considering the memory domain. The result of this formula would be the average result expected for someone with a similar SDI as the patient. By doing a simple cross-multiplication between the ACP memory score and the result of this function, we can get the relative value of the patient when compared with the ND. This value would be, in this example, the memory domain in the CS of the patient, where 50th percentile represents an average performance considering the patients' socio-demographic group in the memory task.

The main challenge here is how to determine the value of weight *W*_*kij*_ in Equation (1) and the value of weight *w*_*i*_ in Equation (2).

#### 2.2.4 Starting with no data

Table 1 depicts each of the NPA instruments' weight in the different cognitive domains and subdomains. As stated above, this NPA instrument aggregation resulted from a focus group session with six neuropsychological assessment and rehabilitation experts. Since most of the selected NPA instrument scores and subscores may contribute to evaluating different cognitive domains and subdomains, the NPA instrument weight was divided for the number of sub-scores it involves and the number of cognitive domains and subdomains it targets.

For example, the MoCA (100%) is a cognitive screening measure that gives us information about general cognitive functioning. Through eight of its subtests (12.5% each), such a multidimensional and comprehensive tool contributes to assessing different domains and subdomains: MoCA calculus (12.5%) contributes to the executive functions assessment in the working memory subdomain (6.25%) and attention in the sustained attention subdomain (6.25%).

For each domain to sum a total of 100%, these values were normalized. As such, if a subdomain has only one NPA tool entry with 50%, it will be normalized to 100%. If no score is given in one or more NPA instruments subdomain, the NeuroAIreh@b system will normalize the existing scores according to the non-normalized values.

**Figure 2:**
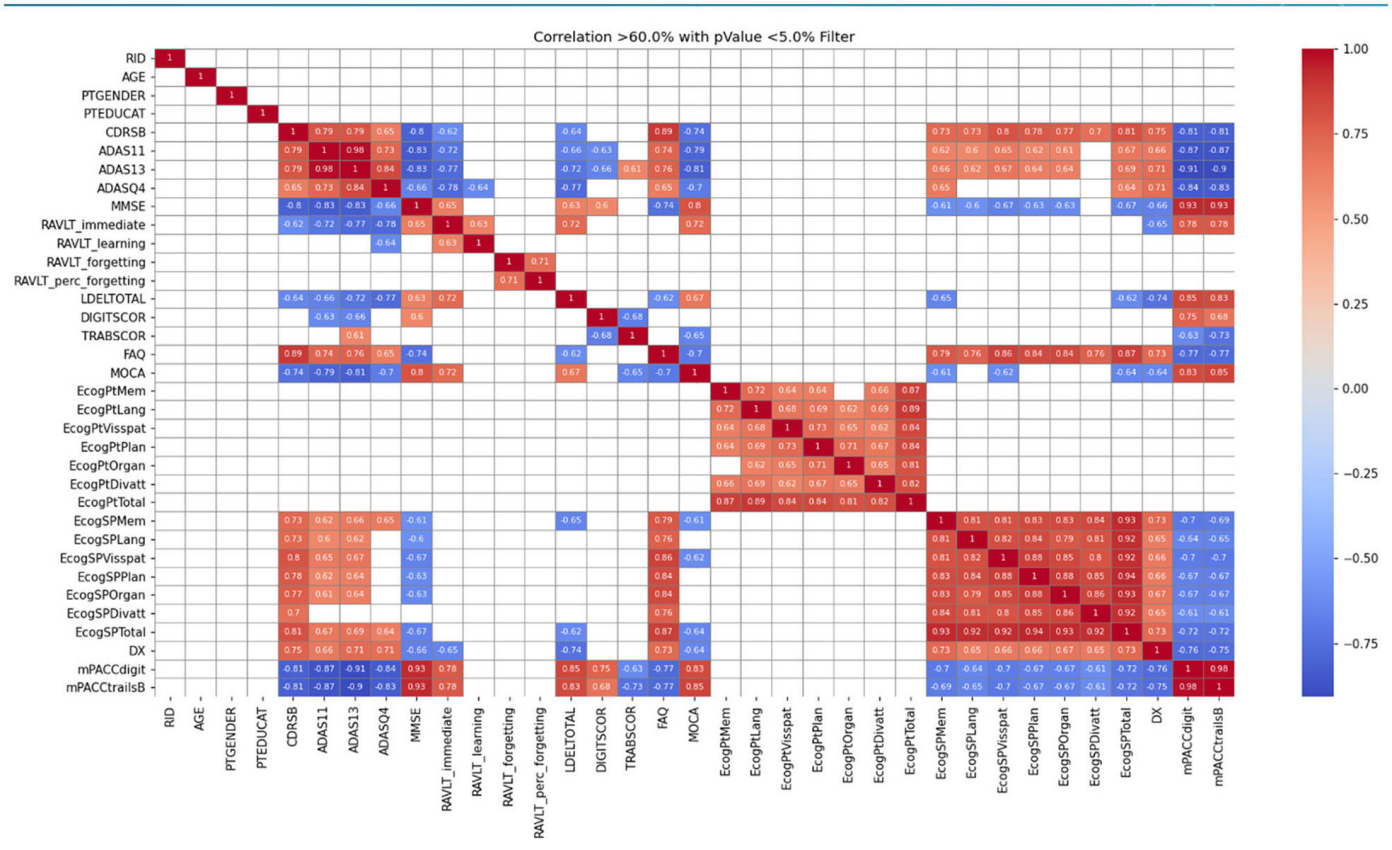
Pearson correlation in the ADNIMERGE table with filter above 60% and *p*-value below 5%.

These empirical values were validated in two different ways: First, we checked if the weighted sum for the aggregation of NPAs validates the basic aggregation rules [see (29), (Chapter 6)]. For the second validation, we compared it with data from other sources, namely the Alzheimer's Disease Neuroimaging Initiative database (ADNI).^1^ The ADNI is a longitudinal multisite observational study of elderly individuals with normal cognition, MCI, and AD. Since it includes a battery of NPAs, we used ML techniques to find correlations between the different NPA instruments and compare them with the empirical correlations, establishing an analogy. To obtain the aggregations for Alzheimer's disease, the following procedure was adopted:

1. A file from the database containing all key tables merged into one was prepared. As an outcome, this file contains the totals from all the key tables and all the assessment results. It includes the diagnosis of each patient, filtered by the NPAs used in NeuroAIreh@.
2. The records were depurated with incomplete data, removing it from the dataset and obtaining a new dataset with around 2200 lines. To clean data sets before creating a model, we have tested the Variance threshold, Pearson Correlation and Analysis of variance.
3. The correlation between tests was checked using the following ML algorithms: Linear Regression, Logistic Regression, Decision Tree, Support Vector Machine, Naïve Bayes, K-nearest neighbors, and Random Forest. Figure 2 shows an example of the correlations obtained. For a full description, see (46). This correlation showed which NPA instruments can assess the exact domains (e.g., memory, depressive symptomatology).

Once we get enough data, we will calibrate *W*_*kij*_ in the Equation (1) and *w*_*i*_ in the Equation (2). For the weight computation, some statistical learning, ML or DL techniques will be applied to NPA instruments data to obtain highly optimal *W* in the Mapping process, such as Principal Component Analysis, random forest or neural network. Besides, if the number of *Dom* and *NPA* grows over time, the system performance may decline, and the data will suffer from high dimensionality. To overcome such problems, feature selection techniques like Principal Component Analysis, LASSO, Ridge or t-distributed Stochastic Neighbor Embedding can be used. These techniques generate highly influential parameters without losing much information.

### 2.3 The training challenge

The training challenge encompasses two different parts: the first one is the development of CTTs to create the CTTs repository, and the second one is the selection and personalization of CTTs given a training objective [see (5) in Figure 1].

The set of CTTs is integrated and managed by a software, the Reh@Sync (47), that is in charge of:

1. Exchanging and managing patient data related to the training sessions with the CTTs.
2. Selection of the ideal CTTs for the provided cognitive profile.
3. Difficulty adaptation during a session and in between sessions.
4. User interface (UI) for patient's interaction with his/her training sessions and CTTs.

#### 2.3.1 The training tasks definition

To identify and select the most relevant ADL-oriented CT content to create the training tasks repository, interviews were conducted with chronic stroke patients (*n* = 15) and neuropsychologists (*n* = 20).

We recruited a sample of 15 stroke participants (nine male, six female) in the community setting, with a mean age of 59.66 (SD = 11.25) and an average of 8.46 (SD = 4.73) years of formal education. Participants were administered the Adults and Older Adults Functional Assessment Inventory (IAFAI) (39), which is a self-report functional incapacity measure that includes both basic (BADL) and instrumental activities of daily living (IADL). We aimed to identify which activities of daily living participants presented impairment—independence with difficulty or dependency—due to cognitive-related factors. Impairment in activities of daily living (ADLs) due to physical or emotional-related factors was not considered. Overall, participants reported more difficulties performing household and advanced IADL, as illustrated in Table 2, due to cognitive-related factors (e.g., attention, memory, problem-solving, mental fatigue). The three most affected IADL domains were conversation and telephone (IADL - Household), comprehension and communication (IADL—Advanced), and use and home security (IADL—Advanced) (48).

**Table 2:** Compromised IADL domains according to community-dwelling stroke patients (*N* = 15).

**Type of IADL**	**IADL domain (items)**	**Items**	**Frequency (%) of patients**
IADL-household	Conversation and telephone use (five items)	Transmit a message	7 (46.67%)
		Understanding what people say	3 (20%)
		Holding a conversation with someone	4 (26.67%)
	Meal preparation (two items)	Cooking a meal	2 (13.33%)
	Home security (six items)	Having contacts for emergencies	1 (6.67%)
		Remembering where important objects are (e.g., keys, documents or money)	5 (33.33%)
		Turn off the stove, oven or iron	1 (6.67%)
IADL-advanced	Comprehension and communication skills (two items)	Telling someone the main aspects of TV news	8 (53.33%)
		Reading and understanding a book or a newspaper	1 (6.67%)
	Health-relation decision making (three items)	Be careful to pick a recipe or buy medication before it runs out	2(13.33%)
		Going to a medical appointment and explaining clearly why	2(13.33%)
		Taking medications as prescribed	4 (26.67%)
	Going out and transportation use (two items)	Going out without getting lost	2 (13.33%)
		Using public transportation when needed	2 (13.33%)
	Leisure time and interpersonal relationships (two items)	Plan and organize something with family or friends	4 (26.67%)
		Continue to perform some usual activities	4 (26.67%)

Concerning the interviews with neuropsychologists, semi-structured interviews were used to inquire about 20 Portuguese professionals with expertise in assessing and rehabilitating stroke patients. These interviews had three main objectives: (a) identify the most common post-stroke cognitive and functional impairments according to their clinical practice; (b) characterize and describe which conventional and/or innovative CR approaches were typically provided following a stroke in the Portuguese clinical setting; and (c) determine guidelines for the development of ICT-based ecologically valid cognitive training interventions (e.g., content, parameters, operationalization procedure, assessment measures) designed for stroke patients. Here, we will focus specifically on objective (c) because it tackles aspects related to the content selection and implementation procedure. As such, the most relevant findings concerning the training content selection and implementation processes will be summarized below (49).

Regarding training content, neuropsychologists stated the importance of designing more ecologically valid cognitive CTTs. They agreed that this could be accomplished by incorporating IADLs' simulations within the CTTs since these activities are known to involve more significant interaction with the social contexts and higher cognitive demands compared to BADLs. In fact, the term cognitive IADLs can be used when referring to everyday or functional cognition, defined as the ability to solve cognitively complex tasks of everyday life in the real world (50). These functional activities typically have a multitasking component and, hence, involve integrating several cognitive processes being engaged simultaneously (51, 52). On that note, the three most mentioned IADLs by the neuropsychologists were meal preparation and cleanup, shopping (e.g., supermarket, restaurant, pharmacy), and financial management, followed by health management and maintenance, driving and community mobility (use of public or private transportation), home management, and functional communication. After specifying the content of the tasks, neuropsychologists were questioned about the tasks' operationalization procedure, i.e., for instance, how did they envision a CTT inspired by the IADL “meal preparation and cleanup” (e.g., what type of instruction would the task have, what would the task goal be)? We found that most neuropsychologists struggled to provide concrete examples regarding this issue; nonetheless, some operationalization proposals for CTTs were given according to their underlying IADL (see Table A1).

**Figure 3:**
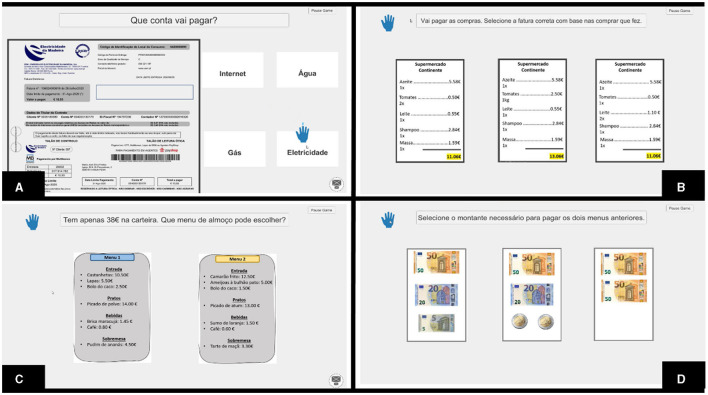
This figure represents four CTTs used in the computerized CT program: **(A)** Analyze the bill task—participants are required to analyze a specific bill (e.g., internet, electricity, gas) and to answer several questions related to that bill (e.g., What is the total cost of the bill?); **(B)** Choose the correct invoice—based on the prices of grocery items shown on a from the previous task, participants were required to select the correct invoice among incorrect invoices. To do so, they needed to retain the information regarding the prices and the number of items and then perform some calculations to estimate the total cost of their purchase; **(C)** Pay the lunch menu(s) task—participants are required to analyze several lunch menus and to select the right menu(s) considering the amount of money available; and **(D)** Pay the lunch—participants must select the correct amount of money needed to pay the previously selected lunch menu(s).

The data gathered from the semi-structured interviews were used to create a preliminary prototype of the digital CTTs using the Musiquence platform (see Figure 3). This platform was initially designed by our team for the cognitive stimulation of people with dementia, capitalizing on music and reminiscence therapy principles (53). Musiquence includes a Game Editor that allows users to develop and customize CTTs based on the users' specificities. Each slide within the Game Editor represents an activity (e.g., quiz 2.0, association, search) that can be completely customized regarding instructions, background images, and response options (53). Each CTT was developed by a psychologist who adjusted task difficulty according to her clinical judgment by manipulating several task parameters (e.g., number of target stimuli, number of distractors, length of the instruments). The CTTs were then organized according to three major themes related to IADLs: functional communication and transportation use, cooking and shopping, and financial management and health-related issues. Subsequently, we have designed a computerized CT program comprising 14 sessions, each lasting 30 min. This program was administered to chronic psychiatric inpatients instead of stroke patients due to restrictions related to the COVID-19 pandemic in accessing the stroke population. The findings of this pilot randomized controlled trial revealed promising preliminary outcomes regarding the impact of the computerized CT program on participants' cognitive and noncognitive domains (54).

#### 2.3.2 The training tasks development process

The design process of the initial set of CTTs consisted of a series of brainstorming sessions among psychologists and developers of the NeuroAIreh@b team. These brainstorming sessions were divided into two parts:

1) First, the information gathered in Section 2.3.1 was analyzed and structured so that the different variables needed for the construction of the digital version of the CTTs were identified.

2) Second, the Braindrawing method was used to design the User Interface, which was also useful for brainstorming the CTTs mechanics (55). Four participants sketched the UI in short design rounds, exchanging the sketches between themselves at the end of each round. At the end of all rounds, results were discussed, and both the UI and the task mechanics were redesigned. Cooking was one of the most mentioned ADLs and, consequently, was the first to be implemented. This task was also used as the basis for deciding on the design and mechanics of the first set of CTTs. The cooking ADL-related tasks addressed three different types of cognitive tasks inspired in our previous work with the Reh@City (11, 12, 56): search and selection (Reh@bSearch), action-sequencing (Reh@bOrganize) and categorization (Reh@bCat) (47).

**Figure 4:**
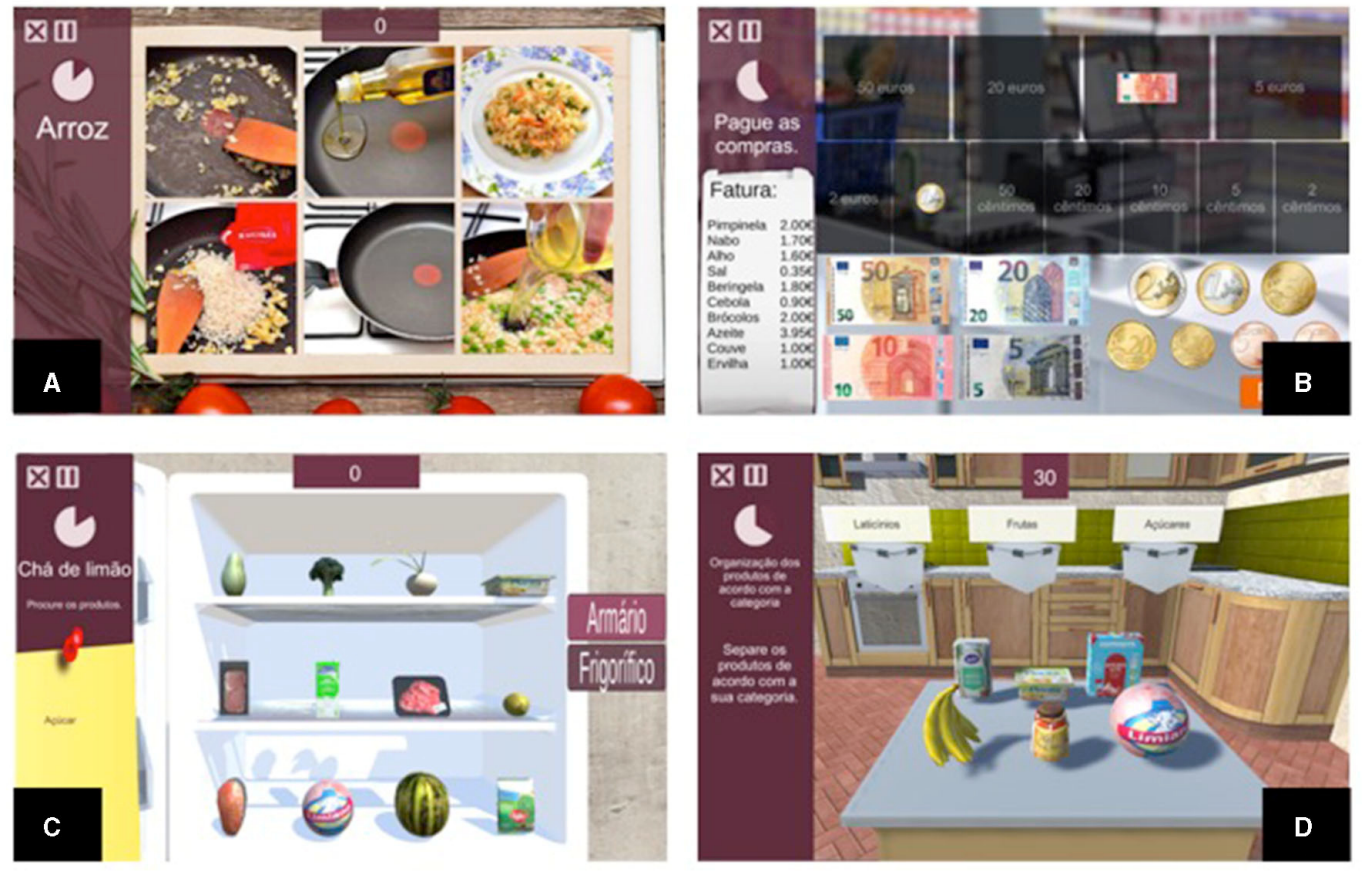
The four Reh@Apps of the NeuroAIreh@b CT platform: **(A)** Reh@Search; **(B)** Reh@Org; **(C)** Reh@Pay; and **(D)** Reh@Cat (47).

In the Reh@bSearch, which consists of a cancellation task, the patient is presented with a list (i.e., shopping list, recipe) and must select the target items (minimum 1; maximum 12) among distractors (maximum elements per section: 20; maximum number of sections: 8) within a time limit in the different sections of a scenario. Reh@bSearch allows the use of different scenarios, such as a supermarket, a kitchen, or a warehouse. The Reh@bOrganize task consists of an action-sequencing task where steps are displayed scrambled to the patient. The patient must organize them in the correct order of execution. This task supports both text and images, with a minimum of 2 and a maximum of 12 steps. In the Reh@bCat, the patient must categorize items (minimum 2; maximum 60) into the correct category container (maximum 4), which can be a fridge or a cabinet, for instance. After being correctly categorized, the item is removed, and a new item to categorize is listed. At the same time, there are yet items to categorize on a list (see Figure 4).

A selection of broad contexts to integrate different tasks was performed. For example, in everyday life, meal preparation and cleanup are commonly performed in the context of a kitchen, and shopping is commonly done in a supermarket. As such, the kitchen contextualized both meal preparation and cleanup activities, and the supermarket contextualized the shopping activity. All the described tasks target several cognitive domains, namely, attention, executive functioning, memory, and language (57). The involvement of each cognitive domain is manipulated according to the goal of the CTT (e.g., to increase memory involvement, the instruction can be removed during task performance; for higher attentional involvement, the number of items can be increased).

There is a process of feedback and reward that is followed by all the tasks, namely: (1) colors and sounds distinguish the correct/incorrect feedback; (2) for each correct action, the patient is rewarded with points and no negative scoring is given on errors; (3) when the established task time ends, the system is prepared to display hints to complete the task and the patient gets half the points; (4) the performance in each task is translated into a percentage that will inform the reward system (lower than 50%—no medal is given, 50–70%—copper medal, 70–90%—silver medal, more than 90%—gold medal).

#### 2.3.3 The training tasks' interdependency

As previously mentioned by neuropsychologists, it is crucial that one task can be transformed into a more complex one. However, previous studies reported that an increase in complexity does not always translate into an increase in performance (58, 59). One way to simplify a complex task is to divide it into smaller steps, and if we take a close look at an ADL, it consists of a series of steps and activities. Let us consider the example of cooking. First, we need to identify the ingredients needed for a determined recipe (interpretation); then, we need to select them from the different places they can be stored in the kitchen (cancellation). Subsequently, we need to perform the needed steps to execute the recipe (action sequencing). Finally, we may need to organize the place by putting the items in their storage place (similar to a categorization task). Although this all happens in the kitchen, we may identify other related activities happening in different scenarios, such as supermarkets. To have the items to cook a specific recipe, we may need to buy them first. Therefore, we identify a specific dependency between activities and scenarios of the same context.

Previous work with the Reh@City did not consider interdependency between activities (12, 56, 57). Nonetheless, we hypothesize that it may be important to improve rehabilitation outcomes since it may increase the ecological validity of the tasks, helping in retaining and transferring decision-making and programming abilities related to complex tasks (59). Therefore, we want to implement this interdependency between the activities of the same context in the NeuroAIreh@b platform. For this, the CTTs content consists of contexts (Main ADLs) and their respective activities (Sub-ADLs) are performed through a cognitive task (for instance, cancellation, action sequencing or categorization).

Since most of the content items that are used to personalize the tasks and adapt difficulty levels are the same for different activities and scenarios, we developed the Daily Life Library (DLL), an Asset Bundle created in Unity 3D^®^(Unity Technologies) where all objects and scenarios, common to all CTTs, are stored. This translates into improving system performance while enabling accessibility to a library of everyday life objects for the current and future NeuroAIreh@b tasks.

### 2.4 Executing the training tasks

#### 2.4.1 The training tasks selection

All the defined ADLs-based CTTs are integrated into the Reh@Sync. All CTTs are independent softwares and can receive the values of their parameters from an external source. Each CTT has its model, which gathers information on the levels of the cognitive domains/subdomains that each CTT trains. This is used by the Reh@Sync to optimize the selection of CTTs [see (5) in Figure 1] in the following way:

1. Reh@Sync receives the cognitive profile of the user, the challenge thresholds, the preferences in terms of ADLs, and the emotional profile as inputs.
2. The personalization manager iterates through all the CTTs, checks their domains' levels and matches them to the CTTs that train the most needed domains of that specific patient. Then, the system returns a list of CTTs ordered by their importance to that patient, where the ones at the top are the ones that target the domains/subdomains that the patient requires to train the most. To calculate this order, we defined a distance (which is a slight variation of Hamming distance) for each domain/subdomain in the following way:
  - 0 if the value of the CS for a domain/subdomain that the patient needs to train is in the range covered by the CTT.
  - *n*, where *n* is the minimal distance between the value of the CS for a domain/subdomain that the patient needs to train and the range covered by the CTT.
  - 10 (the maximal distance value) if the domain/subdomain that the patient needs to train is not covered by the CTT.
3. The content of the activities is filtered by the contexts that match the patient's ADLs with more impairments and the training details (number of sessions and time per session).
4. The Reh@Sync reads each of the selected CTTs and launches it parameterized according to the patient's cognitive profile. The Reh@Sync also oversees the personalization and adaptation of the CTTs to each cognitive profile.

#### 2.4.2 Adaptation during the training session

In our previous studies with the Reh@City (56), each participant was assigned a set of CTTs individually, personalized according to the patient's cognitive profile domains: attention, memory, executive function, and language. This profile was found through the administration of MoCA, with values being converted to a 1–10 scale, with 0.5 intervals. For instance, the maximum value that is possible to achieve on the attention domain of MoCA is 6; this result was then normalized to the Reh@City 1–10 scale, corresponding to the value of 10. The process was similar for the remaining domains: memory, executive function, and language, which can hold the maximum values of 11, 7, and 6, respectively. One additional parameter, the difficulty, was used to adjust the cognitive tasks based on the user performance. The initial value of the difficulty was found by normalizing MoCA's total score to the Reh@City scale.

Then, the intervention consisted of performing task sets. At the end of each set, the difficulty level for the following set of tasks was calculated based on the participant's performance. If the user obtained an average performance lower than 50%, the difficulty was reduced by 0.5 points; if higher than 71%, the difficulty was increased by the same amount; if performance was from 51 to 70%, the difficulty value remained the same. In the NeuroAIreh@b prototype, we implemented this same adaptation method but in a more flexible manner. As such, the neuropsychologist administering the training through NeuroAIreh@b can adjust the minimum and maximum thresholds. This helps the Reh@Sync to learn how to make decisions about when to increase, decrease, or maintain the difficulty. This translates into the following:

- The narrower the thresholds, the higher the number of fluctuations that may occur in terms of difficulty change.
- The wider the thresholds, the lower the number of expected changes in difficulty.
- The lower the maximum threshold, the more difficulty may increase.
- The higher the maximum threshold, the harder it is to have an increase in difficulty.
- The lower the minimum threshold, the more difficulty may decrease.
- The higher the minimum threshold, the harder it is to have a decrease in difficulty.

The Reh@City activities were initially personalized to a specific cognitive profile. Only one parameter, the difficulty level, would change from session to session, considering the overall mean performance of all activities together. In the Reh@Sync, we refine this information in session by evaluating each CTT performance and adjusting the difficulty level for that specific task accordingly. This allows us to tweak the difficulty of the settings to keep the patient in a state of flow (60). It has been proven that people at this level of concentration and immersion are most effective, which is expected to lead to better rehabilitation outcomes.

At the end of a training session, all the performance information is sent to the NeuroAIreh@b server, which estimates a new cognitive profile for the patient that will be sent again to the Reh@Sync, restarting the cycle of CTTs.

### 2.5 The profile dynamics

As we mentioned in Section 2.4.2, the training task adapts the parameters to maintain the performance between a predefined range (e.g., 50–70%). Suppose that a determined parameter of a CTT suffers an increment in its value during the training sessions. In that case, the patient manifests an improvement in his/her CS due to the rehabilitation.

The performances obtained at the end of a training session through the CTTs are used to estimate an intermediary virtual profile that will serve as input to the next session, enabling the CTTs to be adapted to the patient. However, relating the CTTs' difficulty and cognitive domains is not linear because a CTT trains multiple cognitive domains, and it can be difficult to differentiate how much of an obtained performance relates to each specific domain or subdomain.

To help establish this relationship, NeuroAIreh@b creates and maintains a correlation between the parameters of the CTT and the domains/subdomains for each CTT. With these correlations and the performances of all the CTTs in the session, the system summarizes the outcome in a sentence in the language defined in Definition 2.2. This sentence is the input for the profile update. Hence, all combinations of the different domains that could output that specific performance should be considered. By analyzing all the different combinations, we chose the model that displays the lower distance from the previous cognitive profile. The weights of the tasks (CTTs models) are used as a criterion of tiebreaker in case needed [see (24) for the theoretical method]. The new profile obtained will be the input for further training sessions; in other words, it will be used to adapt the CTTs to keep them parameterized to the ideal difficulty level over an iteration of multiple training sessions (which we will call a training program). At the end of each CT program, the patient is reassessed, and the cognitive user profile is compared to the estimated baseline profile.

This comparison will allow us to evaluate the system's performance and see if it performs as expected. The study (61) can help us to understand in which step there was a wrong prediction by the system, given the final result. Once all is done, the loop restarts until the neuropsychologist concludes that the neurorehabilitation process is completed.

## 3 Results

The prototype version of the NeuroAIreh@b has been through a number of clinical validation studies. Since, at the moment, there is a reduced amount of CTTs, a simplification of the NPA aggregation (depicted in Table 1) was performed (Table 3). Instead of assigning weights for each NPA, we considered the minimum and maximum raw scores that could be attained in the different performance-based NPAs. Subsequently, these scores were normalized on a scale of 1–10. Finally, we computed the mean of all the normalized scores within each subdomain to derive a normalized score representing each of the five macro-cognitive domains. This process allowed us to generate the participants' baseline neuropsychological profile, comprising the following macro-domains: general cognition, attention, memory, language and executive functions.

**Table 3:** Cognitive profiling reformulation and simplification.

**General cognition (Min-max** **=** **0–10)**			**MoCA – Total score (Min-max: 0–30)**	
Memory **(Min-max=0-10)**	Immediate	Verbal	FCSRT – Total immediate memory **(Min-max: 0–48)**	
			Visual	ROCFT – 3-minute immediate recall trial **(Min-max: 0–36)**	
	Delayed	Verbal	FCSRT – Total delayed recall **(Min-max: 0–16)**	
			Visual	NA for this study	
Executive functions **(Min-max** **=** **0–10)**	Working memory		Digit symbol coding (WAIS III) **(Min-max: 0–133)**	
	Processing speed		Symbol search (WAIS III) **(Min-max: 0–60)**	Digit symbol coding WAIS III **(0–133)**
	Verbal initiative		Phonemic verbal fluency test **(Min-max: 0–57)**	Semantic verbal fluency test **(Min-max: 0–27)**
	Inhibition		Phonemic verbal fluency test **(Min-max: 0–57)**	Semantic verbal fluency test **(Min-max: 0–27)**
	Visuoconstructive capacity		ROCFT – Copy trial **(Min-max: 0–36)**	
Language **(Min-max** **=** **0–10)**	Expression		Phonemic verbal fluency test **(Min-max: 0–57)**	Semantic verbal fluency test **(Min-max: 0–27)**
	Comprehension		Vocabulary (WAIS-III) **(Min-max: 0-66)**	
Attention **(Min-max** **=** **0–10)**	Divided		Symbol search (WAIS-III) **(Min-max: 0–60)**	
	Sustained		Toulouse-Piéron test – Total score **(Min-max: 0–37.5)**	
Premorbid intelligence **(Min-max** **=** **0–10)**		NA for this study	

An initial pilot study was conducted with ten chronic stroke survivors who were enrolled in a one-month intervention with the prototype version of the NeuroAIreh@b platform (62). The intervention encompassed eight 45-min tablet-based CT sessions. Participants were required to perform four different types of CTTs that were inspired by IADLs (e.g., a cancellation task in the kitchen involving the selection of the correct ingredients necessary to prepare a given recipe, a calculation task in the supermarket consisting of selecting the coins and/or bills necessary to pay for the groceries). The CTTs were implemented using the following reh@apps: Reh@Search (cancellation), Reh@Org (action-sequencing), Reh@Pay (calculation), and Reh@Cat (categorization). In this pilot study, the psychologist was required to parameterize the CTTs manually according to the participant's performance in each iteration, thus modulating task difficulty considering her clinical judgment. Each participant performed each type of CTT for about 11 min. Post-NPAs were conducted to assess the intervention's short-term efficacy. Thus, at post-intervention, there were significant improvements in general cognition, as measured by the MoCA, and in functional abilities, as assessed by the IAFAI. The results from this pilot study suggested that tablet-based CT using the NeuroAIreh@b can lead to immediate short-term benefits in chronic stroke survivors' cognitive functioning and functional abilities. Furthermore, we observed a generalization of training gains to ADLs, potentially attributed to the greater ecological validity of the training content. Importantly, the performance data obtained from this pilot study were used to develop a difficulty progression algorithm to optimize training personalization and adaptation based on participants' neuropsychological profiles and task iterations.

Moreover, a five-week blended neurorehabilitation intervention was conducted with four community-dwelling stroke survivors to evaluate its feasibility, acceptability and preliminary efficacy. The intervention consisted of a total of 15 sessions, delivered between two to three times a week. This program comprised four 90-min in-person sessions, focusing on psychoeducation and compensatory strategies training, and eleven 30-min remote sessions consisting of tablet-based CT with the NeuroAIreh@b platform. Regarding the latter sessions, an additional CTT was incorporated into the platform, specifically designed to target alternating attention. This CTT was implemented through the Reh@Drive app and consisted of driving a car while avoiding road obstacles and collecting gasoline bins. To evaluate the short and long-term impact of the program, a comprehensive neuropsychological assessment was conducted at three different moments: baseline, post-intervention and three-month follow-up.

Firstly, regarding the feasibility of the blended neurorehabilitation program, all participants successfully attended the in-person sessions and completed the prescribed remote sessions, with only minor technical issues (12.5% of technical problems). Consequently, the high training compliance rate highlights the feasibility of the intervention. Secondly, in terms of acceptability, participants reported high levels of satisfaction following the intervention, indicating that the program was meaningful at a cognitive and emotional level. Finally, efficacy-wise, participants demonstrated reliable, differential improvements in several neuropsychological assessment measures immediately after the intervention, some of which were maintained at three-month follow-ups. Furthermore, reliable declines were also observed in two participants, more specifically in processing speed, semantic verbal fluency and visual memory. In addition, no differences were observed concerning participants' changes in goal attainment at post-intervention compared to the baseline. Nonetheless, differences emerged during the three-month follow-up; two participants reported successfully attaining all their rehabilitation goals. On the other hand, only one participant could not achieve any rehabilitation goal.

Overall, our findings provide evidence supporting the feasibility, acceptability and preliminary efficacy of the blended neurorehabilitation. To further validate the tablet-based CT framework (NeuroAIreh@b), we plan to conduct a randomized controlled trial with a larger sample of stroke survivors (63).

## 4 Discussion

Over the last few years, AI techniques have been widely applied in healthcare, raising the discussion of whether, in the future, they would replace health professionals. From our perspective, AI techniques have the potential to complement and enhance the work of health professionals by assisting them in optimizing clinical diagnosis, treatment decision-making and data analysis (64). The ability to learn, self-correct and update the knowledge based on feedback are important AI features that improve its accurateness, thereby reducing assessment and rehabilitation errors that may occur in clinical practice (65). As mentioned above, cognitive deficits rehabilitation is a quite complex process with a series of clinical decisions based on empirical knowledge that would largely benefit from these AI techniques features as a supportive tool.

This work aims to contribute to the advancement of the scientific literature in the area of AI techniques (such as ML and belief revision) applied to the neurorehabilitation of people with cognitive deficits. We believe that the complementarity between AI and neuropsychology creates a virtuous circle advancing both fields' objectives in such an important area as neurorehabilitation. As such, our ultimate goals are 2-fold: (1) provide neuropsychologists with an innovative paradigm to support the clinical decisions in prescribing CT sessions for people affected by cognitive deficits, the NeuroAIreh@b and (2) contribute to a worldwide effort aiming at using AI techniques to improve the management of cognitive deficits associated to stroke, other acquired brain injuries and degenerative disorders (66).

The fact that the NeuroAIreh@b CT tasks are being implemented in VR-based ADLs simulations provides greater ecological validity to the CT (10, 12). Although there is no strong evidence that the use of VR is more beneficial than conventional therapy in cognitive deficits rehabilitation, this technological approach has been demonstrated to be beneficial as complementary to usual care for different reasons: it is more engaging, enables a more intensive training, provides immediate feedback and tasks have greater verisimilitude and validity (7). We believe that the operationalization in VR according to the interviewed patients and neuropsychologists' requirements, will have a positive impact on CT efficacy and transference to everyday-life activities performance, which is the major goal of neurorehabilitation (5).

The NeuroAIreh@b entails an ML component for managing NPA data, which is represented by the ACP, to adapt and personalize the intervention to the patient CP. Although the first CP is made according to the NPA static scores, according to our experts' NPAs aggregation, session-to-session performance in the NeuroAIreh@b CTTs is used by the system to update the profile dynamically. For instance, the patient starts with 7/10 in Memory, but if he/she outperforms, the profile is changed to 7.5/10. The accurate adaptation of the training challenge to the patient performance, together with the use of ecologically valid content, are key elements to enhance engagement, optimize learning, and address specific cognitive deficits more effectively. This has been partially (in our pilot study, adaptation and personalization were manually performed by the psychologist) corroborated by our pilot study, where we concluded that CT with the NeuroAIreh@b platform appears to be beneficial in the chronic phase of stroke, leading to gains in general cognition (MoCA) and functional abilities (IAFAI). These preliminary findings with the prototype version of the NeuroAIreh@b platform were encouraging and suggest the generalization of training gains to the patient's everyday life, which is our main goal and makes our work unique (62).

To strengthen our conclusions and collect data to validate the specific ML algorithms to calibrate the system for profile dynamics, we are performing a randomized controlled trial with stroke patients, which has been approved by the health committees of the involved healthcare institutions (Clinical Trials registration reference: NCT05929287). The intervention with NeuroAIreh@b involves twelve sessions of 30 min during a month. There are two control groups: one performs a paper-and-pencil intervention with the TG (https://neurorehabilitation.github.io/TaskGenerator/), and one is from the waiting list. All participants undergo a baseline NPA to build the initial cognitive profile - the ACP. At the end of the intervention, all participants are re-assessed to measure improvements in the NPA instruments scores. NPA results, together with the data on NeuroAIreh@b and TG performance, will be used to provide real-data evidence to prove the reliability and robustness of the described methodology and models. To verify the maintenance of potential cognitive and functional improvements, participants are submitted to a follow-up assessment at 3 and 6 months post-intervention. Additionally, as we foresee contingencies in accessing large samples of patients, we are already conducting a feasibility study at home. In this study, patients perform a pre and post-neuropsychological assessment at the hospital, but the training sessions are performed at home. This procedure enables the inclusion of participants who do not have the availability or possibility to go to the clinic several times a week. The 10 participants who finished the intervention only reported minor issues and completed the training successfully. After finishing data collection, we will establish partnerships with other Portuguese hospitals and clinics to enable a more significant number of participants.

## 5 Limitations

Since this work refers to presenting a methodology that has not been completely validated with an RCT, there are important limitations to be acknowledged. First, there is the fact that we are starting with no data, and the ACP algorithms are only learned through reasoning by analogy from similar existing work with the ADNI database. Using algorithms that are based on Alzheimer's Disease patients' data might not apply equally to all persons with cognitive disorders. For instance, it is expected that the evolution of the CS of acquired brain injury patients (namely, stroke and acquired brain injury) is different from the degenerative disorder patients (namely, Alzheimer's Disease). For the ACP to be updated during an intervention, the AI system needs to learn with data from patients' performance in undergoing future clinical RCTs. The pending verification/validation of the specific ML algorithms to calibrate the system for profile dynamics, which depends on collecting a considerable amount of data, is one of the limitations of our present work and one of the main challenges we face in our future work. Second, we use the MoCA subtests to account for specific domains of cognition in the NPA instruments aggregation to create the ACP. Since the MoCA is a screening tool and does not comprehensively assess specific cognitive domains, we may lack precision in our approach, especially in the cognitive domains that mainly rely on MoCA subdomain assessment results. Third, in this phase, the conceptualization and selection of the cognitive domains that are trained with each CTT are assumed and selected based on experts' opinions and experience and are still not empirically validated. Due to this inherent growing complexity of underlying models and algorithms in this methodology, AI appears here as a “black box” because the internal learning processes, as well as the resulting models, will not be entirely comprehensible (67). In other words, as we collect more data, we may not be able to understand what the cognitive constructs involved in each CTT are and how they are selected to match each ACP. Fourth, we make assumptions on what needs to be prioritized with regard to the CTTs to be selected based on numeric data from the ACP, that is, by its turn, based on our NPA aggregation (an implicitly made relationship between different types of cognitive functions and different levels of test results and their interaction). Additionally, we propose specific CTTs and implicitly assume an inherent cognitive profile. Again, in this phase, we cannot warrant that this approach is more clinically effective than any other that could be used here. The NeuroAIReh@b methodology still needs to be validated, and then, as future work, we could compare it with a different personalization and adaptation approach.

## Data availability statement

The original contributions presented in the study are included in the article/Supplementary material, further inquiries can be directed to the corresponding authors.

## Ethics statement

The studies involving humans were approved by Comissão de Ética do Serviço de Saúde da Região Autónoma da Madeira, Comissão de Ética da Casa de Saúde Câmara Pestana, and Comissão de Ética do Centro Hospitalar e Universitário de Coimbra. The studies were conducted in accordance with the local legislation and institutional requirements. The participants provided their written informed consent to participate in this study. Written informed consent was obtained from the individual(s) for the publication of any potentially identifiable images or data included in this article.

## Author contributions

AF: Methodology, Supervision, Writing – original draft, Writing – review & editing, Conceptualization, Validation. YA: Conceptualization, Formal analysis, Methodology, Writing – original draft. DB: Software, Investigation, Writing – original draft. JC: Conceptualization, Methodology, Validation, Writing – original draft, Writing – review & editing, Investigation. MC: Supervision, Conceptualization, Writing – review & editing. LF: Software, Conceptualization, Writing – original draft. AM: Validation, Writing – original draft. TP: Software, Writing – original draft, Conceptualization, Formal analysis, Investigation, Methodology. PR: Data curation, Formal analysis, Writing – original draft. MSp: Investigation, Validation, Writing – original draft. MV: Conceptualization, Supervision, Writing – review & editing. SB: Conceptualization, Funding acquisition, Methodology, Resources, Supervision, Writing – review & editing. MSi: Conceptualization, Funding acquisition, Resources, Supervision, Writing – review & editing. EF: Conceptualization, Formal analysis, Funding acquisition, Investigation, Methodology, Project administration, Resources, Supervision, Writing – original draft, Writing – review & editing.

## Footnotes

^1^ http://adni.loni.usc.edu
