# Supplementary material for: NeuroAIreh@b: an artificial intelligence-based methodology for personalized and adaptive neurorehabilitation
Source: Front Neurol. 2024 Jan 19;14:1258323. doi: 10.3389/fneur.2023.1258323 (PMC10846403; doi:10.3389/fneur.2023.1258323)
Supplement: Supplementary file 1 [file Table_1.pdf]

APPENDIX 1: TABLES

IADL	Cognitive training task operationalization description
Meal preparation and cleanup	- Identifying the ingredients and utensils required to prepare a specific meal (e.g., attention task, where the person must select the target ingredients and utensils among other ingredients and objects (distractors));<br>- Planning the different steps necessary to prepare a meal (e.g., action sequencing task, in which the person must organize a set of images that depict a particular meal, in the correct order);<br>- Making decisions regarding which meal to prepare according to specific instructions (e.g., problem-solving task, where the person must read a recipe book and target which meal to prepare among a pool of distractor meals, considering the ingredients available in the fridge).
Shopping	- Making a shopping list based on the need to cook a particular meal (e.g., attention task, where the person must analyze a recipe, target the ingredients already available in the kitchen and write down the missing ingredients that must be bought in the supermarket);<br>- Identifying the products written on the shopping list that are displayed on the supermarket shelves (e.g., attention task, where the person must select the target products among distractors).
Financial management	- Prioritizing expenses/bills (e.g., planning, and problem-solving task, in which the person must arrange the bills in a chronological order according to their due date, i.e., identify the bills that should be paid first and so forth);<br>- Analysis and interpretation of invoices (e.g., reading comprehension task, where the person must select the correct response to a given question (e.g., "What is the electricity bill due date?") among several distractor responses);<br>- Performing payments (e.g., calculation tasks, in which the person must perform arithmetic operations, such as addition, subtraction, and multiplication when paying shopping bills).
Health management and maintenance	- Organizing medications in the weekly medication organizer according to a medical prescription (e.g., attention and planning task, where the person needs to arrange the different pills in the correct time schedule and dosage by dragging and dropping them into the correct compartment);<br>- Identifying the correct medication schedule and dosage (e.g., memory tasks with two different stages, namely a retention phase (e.g., "Try to memorize when to take medications A and B, and their correct dosage"), followed by a second phase consisting of a delayed recall task involving a voice recognition or multiple-choice recognition task (e.g., "Indicate at what time medication A and B should be taken, and in what dosage?"));<br>- Buying medication at the local pharmacy (e.g., action sequencing task using images, where the person needs to select the images for the correct order, i.e., identify the first image, the second image, and so forth).

Provisional

IADL	Cognitive training task operationalization description
Driving and community mobility	- Identifying and selecting the correct public transportation according to a specific target location (e.g., attention and problem-solving task, where the person must analyze several bus routes and select the correct one among a set of distractor routes);<br>- Estimating the time needed to arrive at a given target location (e.g., calculation task, where the person is presented with a map containing estimations of the required time to travel to different locations and must indicate the number of minutes that will take to go from a target location to another (e.g., A to B));<br>- Finding alternative responses/solutions to a given spatial orientation/navigation task (e.g., problem-solving, and cognitive flexibility task, in which the person is presented with a particular problem (e.g., "Imagine that you have to be at location A in around 10 minutes. You missed the bus and the next one will come in 30 minutes. How can you be at location A on time?"), and must select the correct alternative among a set of incorrect ones ("A-By taking a taxi (duration: 5 minutes); B-By walking (duration: 15 minutes), etc.")).
Home establishment and management	- Organizing the closet (e.g., categorization task, in which the person needs to organize the clothes according to specific categories, such as the color, the season and the type of garment);<br>- Placing the dishes in the dishwasher (e.g., planning task, where the person must put the dishes in the correct dishwasher compartment according to the size of the dish, the available space in the compartment, etc.);<br>- Setting the table (e.g., action sequencing task, where the person needs to organize a set of images that illustrate the action of setting the table in the correct order).
Functional communication	- Transmit a message/information after a specific time interval (e.g., memory task involving two stages; the retention phase (e.g., "Please do not forget to turn off the oven after 10 minutes"), followed by a delayed recall phase conducted via a voice recognition or multiple-choice recognition task (e.g., "Identify, among the available answers, the content of the message that you read earlier);<br>- Analysis and interpretation of news (e.g., memory task comprising two stages; the retention phase (e.g., "Please read the following article in the newspaper and try to remember its content"), followed by a delayed recall phase conducted via voice recognition or multiple-choice recognition tasks).

Table 1 Examples of cognitive training tasks operationalization proposals according to each instrumental activity of daily living.

Provisional
